# Field‐Driven Active Colloids as Distributed Micromachines: Bridging Physics, Control, and Function Across Length Scales

**DOI:** 10.1002/advs.78065

**Published:** 2026-09-30

**Authors:** Ruchi Patel, Bhuvnesh Bharti

**Affiliations:** ^1^ Cain Department of Chemical Engineering Louisiana State University Baton Rouge Louisiana USA

**Keywords:** active matter, collective behavior, colloid, control reconfiguration, electric field, magnetic field, nanotechnology, physics, propulsion, task oriented

## Abstract

Field‐driven active colloids offer a route to microscale machines whose behavior can be programmed by external electric and magnetic fields. At the micron scale, thermal fluctuations, hydrodynamic interactions, interparticle forces, and field‐mediated actuation combine to connect active matter physics with control and function. In this Perspective, we frame field‐driven active colloids as distributed micromachines, where power delivery, sensing, control, and function are distributed across particles, applied fields, imaging modalities, and feedback architectures. Electric and magnetic fields enable programmable propulsion, trajectory regulation, collective interactions, and dynamic reconfiguration, but practical deployment remains limited by energy delivery, material compatibility, and feedback constraints in complex environments. We emphasize that progress should be evaluated not only by motion, but by controlling task‐relevant variables that determine successful localization, transport, capture, treatment, release, assembly, and reconfiguration. Closing the loop between sensing, AI/ML, computation, and field‐based actuation will be essential for advancing field‐driven colloids toward adaptive, task‐oriented micromachines.

## Graphical abstract

Field‐driven active colloids are framed as distributed micromachines in which externally supplied power, sensing, and control regulate task‐relevant states. By closing the loop between particle behavior, imaging, computation, and electric or magnetic actuation, these systems can progress from programmable motion toward adaptive functional outcomes, including navigation, transport, capture, treatment, and collective reconfiguration.

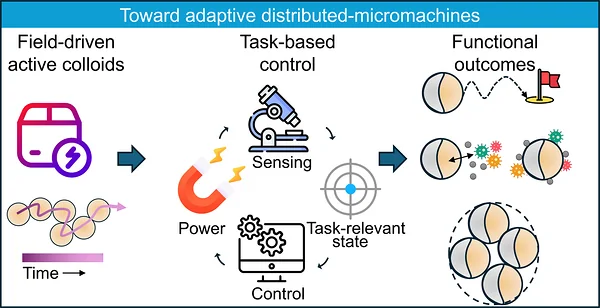

## 1 Introduction

The ability to achieve controlled and functional behavior across length scales remains one of the central challenges in designing dynamically reconfigurable materials [1, 2, 3]. At molecular and nanometer length scales, material transport and organization are often dominated by thermal fluctuations, viscous dissipation, and stochastic collisions, making persistent directed motion and programmable structural reconfiguration inherently difficult to achieve [4, 5, 6]. In contrast, macroscopic materials often operate in a more deterministic, inertia‐dominated regime, where dynamics, assembly, and function can be controlled externally with high precision [7]. Although these regimes span different length scales [8] and are governed by distinct physical mechanisms, knowledge transfer across them can help bridge molecular‐scale design, microscale control, and macroscale actuation. Bridging this divide is therefore essential for transforming fluctuation‐dominated molecular and nanoscale materials into controllable, functional, and dynamically reconfigurable matter.

Colloids occupy an intermediate size regime that connects fluctuation‐dominated molecular‐ and nanoscale materials with externally controlled macroscopic machines [9]. At length scales spanning roughly 0.1–10 µm, colloidal dynamics emerge from the interplay among Brownian fluctuations, hydrodynamic interactions, interparticle forces, and externally applied fields, such that neither stochasticity nor deterministic control fully dominates behavior [9, 10, 11]. This coexistence makes colloids a powerful platform for studying how control and fluctuations compete: they are large enough to be directly visualized, manipulated, and dynamically programmed in real time, yet small enough for fluctuations to remain intrinsically coupled to motion, transport, and collective organization [12, 13, 14, 15]. Consequently, colloids offer a unique platform for bridging physics, control, and function across multiple length and time scales by combining externally directed behavior with fluctuation‐mediated dynamics.

Active colloids are microscale particles that propel through fluids by generating asymmetric force distributions through chemical activity, external fields, or engineered particle anisotropy. These particles convert energy into directed motion, dynamic interactions, and adaptive collective behavior far from equilibrium [15, 16, 17, 18, 19] (Figure 1). Field‐driven active colloids are a distinct subset of active matter in which particles are driven by an external field [12, 16, 17, 19, 20, 21, 22]. Although optical fields provide tools for colloidal manipulation and actuation, this Perspective focuses on active colloids driven by electric and magnetic fields, where field strength, frequency, and temporal modulation can dynamically regulate propulsion, trajectories, and interactions. These field‐energized particles provide a route toward programmable microscale behavior by combining externally directed actuation with fluctuation‐modulated dynamics [15, 17, 19, 20]. In recent years, several sophisticated approaches have been developed for controlling these active particles through field modulation, real‐time imaging, feedback control, and collective interaction design, thus moving beyond superdiffusive active motion toward externally programmable micromachines [23, 24, 25, 26, 27, 28, 29].

**FIGURE 1:**
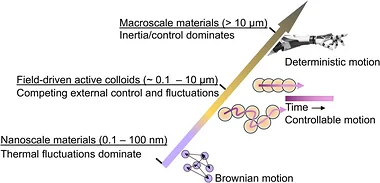
Field‐driven active colloids occupy an intermediate size regime between fluctuation‐dominated nanoscale materials and more deterministic macroscopic materials. At nanometer length scales, thermal fluctuations dominate motion and transport, whereas macroscopic materials often exhibit inertia‐ or control‐dominated dynamics. Field‐driven active colloids (∼0.1–10 µm in size) operate between these limits, where Brownian fluctuations, hydrodynamic interactions, interparticle forces, and applied electric or magnetic fields act together. This coexistence of fluctuations and external actuations enables controllable motion, collective dynamics, and feedback‐guided behavior, positioning field‐driven active colloids as a platform for bridging colloidal physics with robotic function.

In this Perspective, we frame field‐driven active colloids as a platform for bridging physics, control, and function across length scales by drawing conceptual parallels between active colloids and macroscopic robots. We first discuss how active colloid behavior emerges from the competition between inherent fluctuations and external control. We then review strategies for programming particle motion and collective dynamics using electric and magnetic fields. Next, we highlight recent efforts to achieve higher levels of control through real‐time feedback and externally guided actuation. Finally, we discuss challenges and opportunities for the next decade, including feedback architectures, artificial intelligence/machine learning (AI/ML)‐assisted control, and data‐driven models for enabling increasingly sophisticated and task‐oriented functions.

The distributed‐micromachine framework proposed here is not intended simply as a microscale translation of conventional robotic architectures. Here, “distributed” means that power transduction, sensing, control, and task execution are not colocated within a single autonomous particle but are partitioned across the particles, applied fields, surrounding environment, and external feedback system. Information and control authority are likewise decentralized, with collective function emerging from globally applied inputs and local particle–particle and particle‐environment interactions. The distinguishing feature of distributed‐micromachines is a fundamental disparity between the high dimensionality of the physical system and the comparatively low dimensionality of its control interface. A population of field‐driven colloids contains many microscopic degrees of freedom associated with particle position, orientation, interactions, interfacial properties, and local environment. These degrees of freedom are manipulated through a small number of globally applied field parameters, such as field amplitude, frequency, phase, or gradient. However, only a subset of the instantaneous particle and environmental state is experimentally observable. The central design problem is therefore not necessarily to independently control every particle, but to identify a reduced set of task‐relevant variables that can be both observed and regulated using the available field inputs. This distinction connects active‐matter descriptions of reduced collective states with microrobotic control and provides a basis for evaluating whether increasingly complex particle dynamics actually translate into controllable function. The central message of this Perspective therefore is that field‐driven active colloids represent distributed micromachines in which particles, fields, imaging, feedback, and task‐specific components collectively provide the functional architecture. In our view, a successful design of distributed micromachine requires matching the available sensing and actuation channels to the reduced set of variables that determine task completion.

## 2 From Robotics Modules to Microscale Functionality

In a simplified form, robotic functionality can be understood as emerging through the coordinated coupling of four interconnected modules: power, sensing, control, and function [30, 31] (Figure 2A). A power source provides the energy required for all operations, while sensing modules acquire information about the surrounding environment and the state of the robot. This information is subsequently processed through a control architecture that determines how the robot should respond, enabling targeted actions through actuation, navigation, or manipulation through a function module. The integration and continuous communication among these modules allow robotic platforms to adapt their behaviors in dynamically changing environments. Although modern robots involve more complex hardware, software, communication, and decision‐making architectures, this simplified modular description provides a useful framework for identifying the basic capabilities required for task‐oriented behavior at the macroscopic length scale. For microscale robots and active colloids, this framework is especially useful because it clarifies which robotic capabilities can be embedded within the particle, and which need to be externally supplied.

**FIGURE 2:**
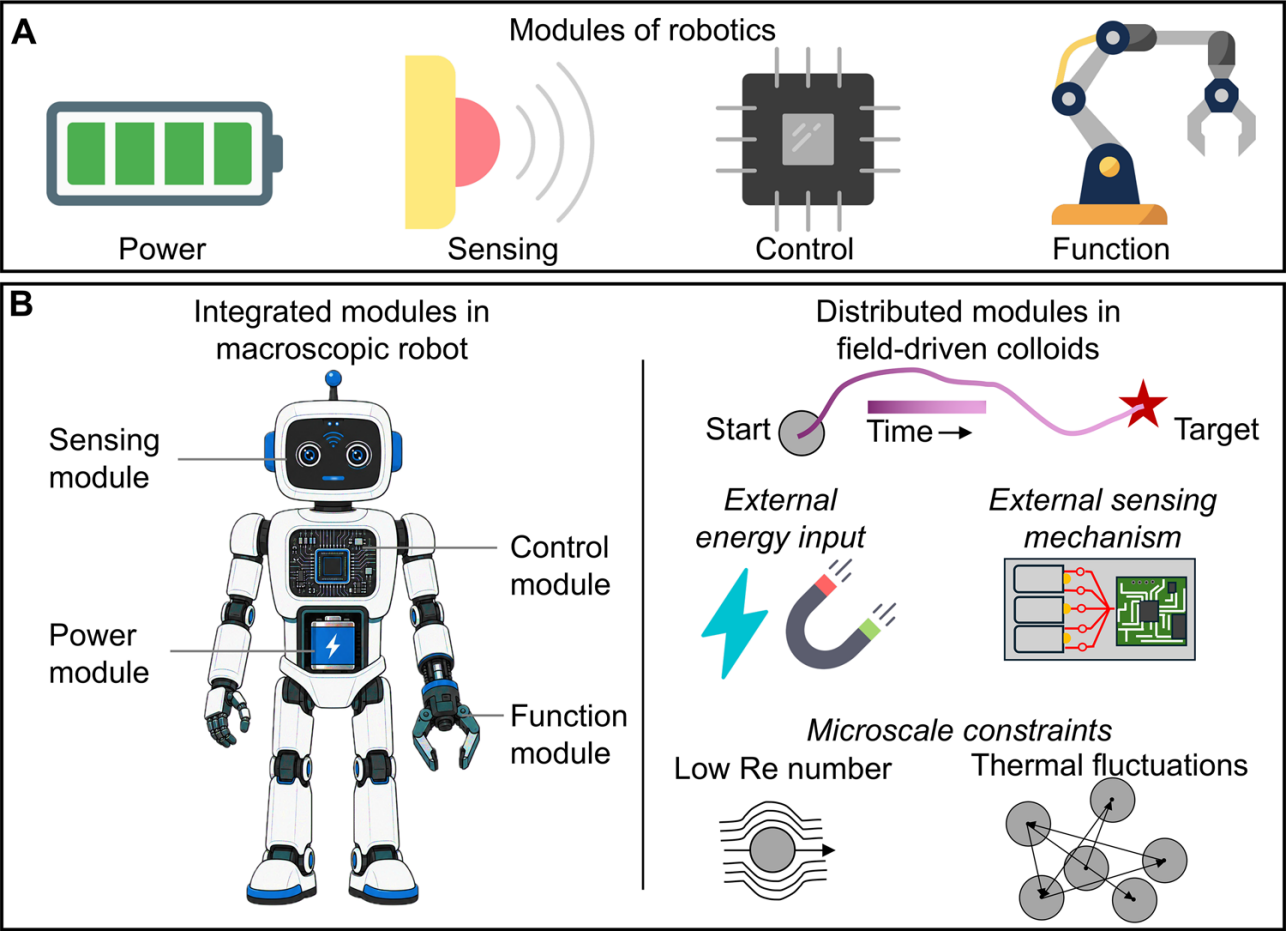
(A) Robotic functionality can be described simply through the coordinated coupling of four interconnected modules: power delivery, sensing, control, and function. (B) Macroscopic robots achieve adaptive and task‐oriented behavior by integrating modules within a single robotic platform. At microscopic length scales, direct integration becomes difficult because onboard power delivery, sensing, and computation are strongly constrained, while low Reynolds number motion and thermal fluctuations perturb trajectories and orientations. Field‐driven active colloids therefore operate through a distributed robotics architecture, in which external fields supply energy, imaging provides sensing, feedback algorithms impose control, and particle motion or collective reconfiguration produces function. The schematic of the humanoid robot shown in B was generated using ChatGPT version 5.5 on June 25, 2026.

Macroscopic robots achieve adaptive and task‐oriented functionality by physically integrating all four modules within a single robotic platform (Figure 2B). Embedded power supplies, onboard sensors, processors, and actuators continuously interact through real‐time feedback loops, allowing precise control over motion, navigation, and task execution. However, directly translating this architecture to microscopic length scales remains fundamentally challenging. At the microscale, the miniaturization of onboard power sources, sensing components, and computational architectures becomes increasingly difficult, while viscous forces dominate inertia and thermal fluctuations continuously perturb particle trajectories and orientations [30, 32, 33, 34, 35]. As a result, the integrated robotic architectures characteristic of macroscopic robots cannot be directly replicated at microscopic scales, requiring alternative approaches for achieving controllable microscale functionality.

Biological and biohybrid microswimmers can already perform many functions associated with microscale motility, including self‐propulsion, environmental response, and adaptive changes in motion [32, 36]. However, their behavior remains difficult to program with the spatial and temporal precision needed for generalizable robotic function. Nevertheless, their dynamics provide important design principles by highlighting the need to couple energy conversion, sensing, response, and task execution at microscopic length scales. These lessons motivate the development of fully synthetic micromachines in which propulsion, navigation, and collective behavior can be regulated externally. In field‐driven active colloids, the robotic modules instead become distributed rather than physically integrated within the particle itself (Figure 2B). External electric and magnetic fields provide continuous energy input for propulsion and motion, whereas imaging methods serve as sensing pathways. Feedback algorithms and externally imposed field modulation then act as control architectures that guide particle behavior in space and time [17, 19]. The resulting particle motion and collective dynamics enable early functional capabilities including transport, navigation, and reconfiguration.

The central premise of this Perspective is that field‐driven active colloids are best viewed through a *distributed robotics architecture*. Rather than being treated as autonomously moving particles, they can be understood as externally programmable micromachines whose behaviors can be guided, regulated, and reconfigured in space and time. Externally imposed fields and feedback architectures provide a route toward artificially embedding adaptive and feedback‐responsive behaviors into microscale particles that inherently lack onboard sensing and computational modules. The following sections build on this framework by discussing how electric and magnetic fields are being leveraged not only to achieve propulsion, but also to create controllable and functional active colloidal matter.

An important consequence of this distributed architecture is that microscopic controllability need not be equivalent to functional controllability. A field may simultaneously act on thousands of particles while providing only a few independently tunable inputs, making particle‐by‐particle control intrinsically difficult. However, many useful functions are lower dimensional than the particle dynamics itself. For example, navigation may require control of a particle or swarm centroid, pollutant removal may depend primarily on adsorption state, and reconfigurable materials may be characterized through order parameters, cluster size, or spatial density rather than the position of every constituent particle. We therefore propose a task‐level design principle: *the relevant state of a distributed micromachine should be the minimum set of measurable variables sufficient to predict and regulate task completion*. From this perspective, the central question becomes not whether every microscopic degree of freedom can be controlled, but whether the available field inputs can reproducibly perturb the variables that determine function and whether those variables can be measured with sufficient fidelity to support feedback control.

## 3 Programming Motion: From Symmetry Breaking to Field‐Mediated Control

Programmable motion represents the first major step in translating the distributed robotics framework into field‐driven active colloids [41]. Early chemically powered Janus colloids generated directed transport by creating asymmetric chemical gradients around the particle surface, enabling propulsion through phoretic effects such as self‐diffusiophoresis and self‐electrophoresis [16, 42, 43, 44] (Figure 3A). Such particles established the foundations of synthetic active matter by demonstrating that broken symmetry can convert local energy dissipation into persistent motion. However, the resulting trajectories remained strongly influenced by fluctuations and were difficult to dynamically modulate after particle fabrication. Field‐driven active colloids address this limitation by shifting energy input and trajectory regulation from the particle to externally applied electric and magnetic fields. In alternating current (AC) electric fields, induced‐charge electrophoresis (ICEP) first enabled controlled linear propulsion of Janus colloids through asymmetric electrohydrodynamic flows generated around metallodielectric particles [45]. Subsequent studies demonstrated that precisely tuning the particle asymmetry and patch geometry could change the propulsion mode itself, enabling transitions from linear to helical trajectories with controllable pitch, diameter, handedness, and translational velocity (Figure 3B). Importantly, both intrinsic particle properties, such as particle and patch size and shape, and extrinsic field parameters, including field strength and frequency, can dynamically regulate the resulting kinematics, thereby transforming propulsion into a continuously programmable process [37, 46]. Numerous subsequent studies have further expanded this framework by demonstrating tunable electrokinetic propulsion, steering, and trajectory control across a wide range of active colloids. The central physical control problem is therefore to determine how particle form factor, surface polarizability, double‐layer charging, and AC‐field timescales together dictate propulsion mode, speed, and trajectory.

**FIGURE 3:**
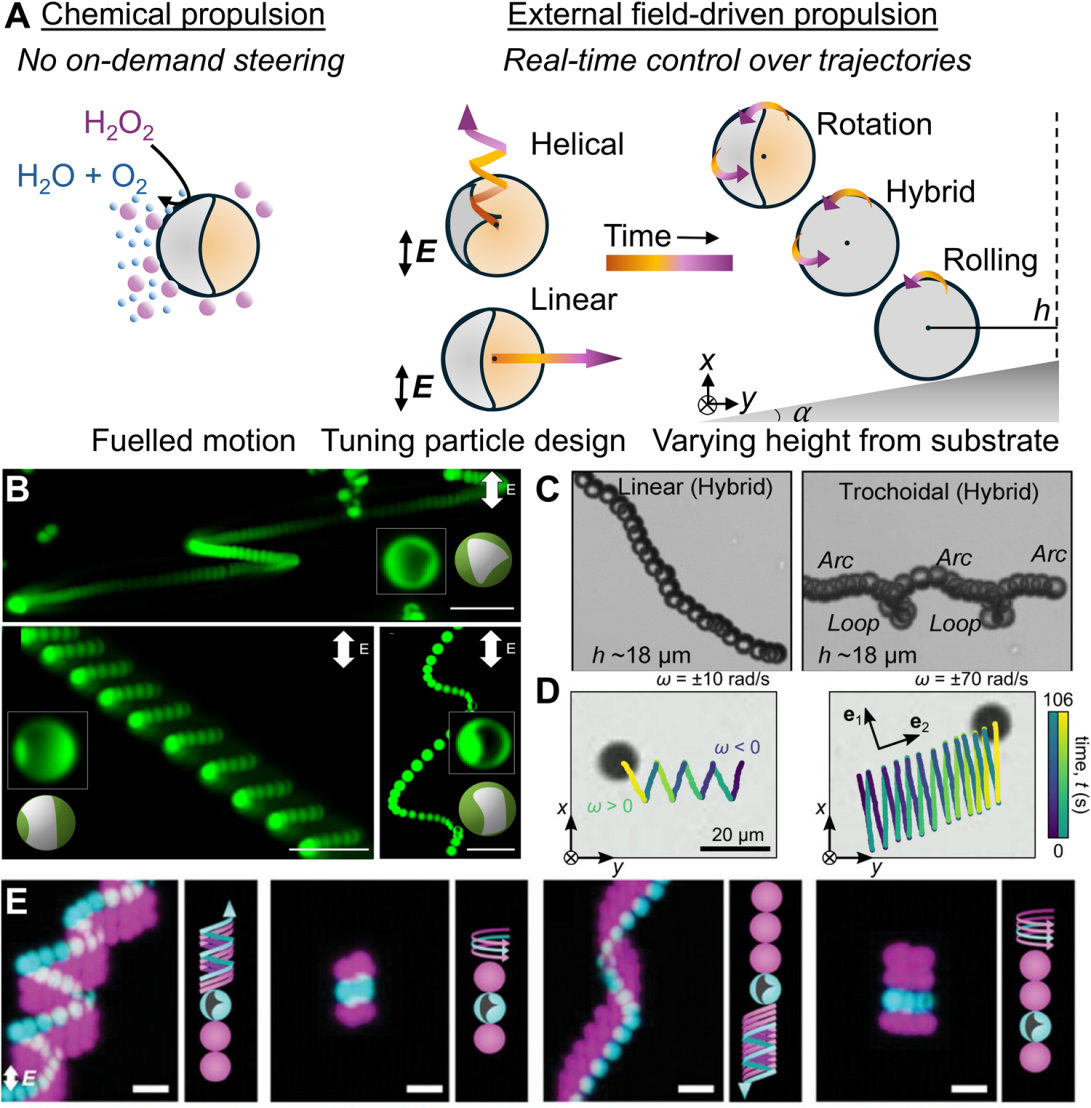
(A) Comparison between classical chemically fueled propulsion and externally controlled field‐driven motion. Classical chemical propulsion generates superdiffusive motion, whereas external electric and magnetic fields enable real‐time control over trajectories and propulsion states of the colloidal particles. (B) Electric field‐driven active colloids exhibit symmetry‐dependent propulsion modes, including helical and linear trajectories, under applied AC electric fields. (C, D) Magnetically driven active colloids exhibit controllable rolling, arc‐like, and trochoidal trajectories, where the alternating magnetic field allows for continuous rotation of the particles above a substrate. (E) Trajectories of co‐assembled active and passive colloids formed through non‐reciprocal interactions between motile active particles and Brownian passive particles show dynamic reconfiguration. The images in B and E are overlays of the individual frames of the microscopic videos. Panel B is adapted from reference [37] (CC BY 4.0), C from reference [38] (Copyright 2026 Royal Society of Chemistry), D from reference [39] (Copyright 2024 American Chemical Society), and E from reference [40] (Copyright 2025 Wiley‐VCH).

Magnetic actuation further demonstrates how external fields can transform particle motion into programmable navigation by coupling field‐controlled rotation with translation, boundaries, and environmental constraints. Time‐varying magnetic fields have been used to drive rolling of particles near surfaces and rotation away from substrates [47, 48, 49, 50]. Recent studies have revealed hybrid dynamical regimes in which translational and rotational motion coexist at intermediate particle‐substrate [51] separations, *h* producing field frequency‐dependent linear, arc‐like, and trochoidal trajectories [38] (Figure 3C). Transitions between phase‐locked and phase‐slip rotational states provide a mechanism for switching between distinct trajectory classes, which can be dynamically reconfigured through time‐varying magnetic fields. Additional studies further demonstrate that not only field frequency, but also environmental parameters such as substrate inclination and confinement geometry, can continuously tune particle kinematics and directional transport [39] (Figure 3D). Collectively, these studies illustrate how field‐driven colloids enable programmable motion through simultaneous control over particle properties, field characteristics, and boundary‐mediated interactions. Such dynamically tunable trajectories and adaptive kinematics position field‐driven active colloids as promising platforms for microscale navigation, cargo transport, environmental exploration, and reconfigurable microrobotic functionality in complex environments. Here, the scientific challenge is to determine how magnetic torque, viscous drag, Brownian rotation, substrate coupling, and dynamics at step‐out field frequency collectively define the accessible trajectory space. These particle‐dependent response functions also provide a means of partially overcoming the limitations of global actuation. Particles with different polarization spectra, magnetic moments, shapes, rotational mobilities, or characteristic response frequencies can respond differently to the same imposed field. We refer to this strategy as “physical multiplexing,” in which particle properties encode distinct transfer functions between a common global input and particle‐level motion or interaction. From a distributed micromachine‐design perspective, particle heterogeneity can therefore be engineered not only to maximize propulsion, but also to increase the number of distinguishable responses accessible through a limited set of field inputs.

Field‐mediated control also extends programmable motion from individual trajectories to collective states in which interactions, assembly/organization, and reconfiguration are externally regulated. In AC electric fields, the interplay between dipolar attractions and ICEP propulsion generates non‐reciprocal interactions capable of driving the formation of metastable active clusters with dynamically tunable trajectories, fluctuations, and reconfiguration pathways [40] (Figure 3E). Depending on the balance between propulsion and field‐induced interactions, these active assemblies can transition between translational, rotational, and helical collective states while continuously reorganizing in space and time. Similarly, magnetic microswimmers assembled from superparamagnetic particles subjected to eccentric magnetic fields exhibit non‐reciprocal body deformations that couple directly to controllable swimming dynamics, enabling externally programmable collective motion and directional transport [52]. More broadly, numerous studies have demonstrated that programmable collective dynamics can emerge from carefully designed interactions between propulsion, external fields, hydrodynamic coupling, and particle geometry, enabling active colloidal assemblies that continuously adapt and reconfigure under external control [13, 14, 40, 52]. These advances extend programmable single‐particle dynamics toward reconfigurable collective microbots. From this perspective, field‐driven colloids provide a route not only toward controllable active matter, but also toward reconfigurable micromachines whose motion, non‐reciprocal interactions, and collective functionality can be programmed in real time through externally imposed control landscapes. A key challenge is therefore to establish *design rules* that connect particle‐level propulsion, interaction anisotropy, and field‐mediated coupling to collective observables such as cluster lifetime, reconfiguration rate, collective velocity, and orientational order.

## 4 Constraints Shaping Field‐Driven Active Colloids

Electric and magnetic fields provide a route for regulating particle trajectories, interactions, and collective dynamics in real time, positioning field‐driven colloids as distributed micromachines [15, 17, 19, 53, 54]. Considerable progress has been made in programming propulsion, steering, and reconfiguration through externally imposed fields [26, 28, 37, 38, 39, 40, 52, 55, 56, 57]. However, translation beyond proof‐of‐concept demonstrations remains limited by practical constraints that determine whether controlled motion can be sustained in application‐relevant conditions. These constraints arise because field‐driven motion is not governed by the particle alone; it depends on interactions among the applied field, particle polarization, interfacial charge, hydrodynamic drag, medium conductivity, boundaries, and feedback latency. As a result, actuation that is robust in dilute, transparent, and well‐defined laboratory media can become inefficient or unpredictable in crowded biological environments, porous matrices, and other complex fluids. Here, we identify three key bottlenecks that currently limit the translation of field‐driven colloids into functional micromachines: (1) energy delivery, (2) material constraints, and (3) sensing and imaging (Figure 4). Recognizing these constraints as design requirements provides a pathway for moving field‐driven colloids beyond proof‐of‐concept demonstrations toward task‐oriented micromachines. To translate these bottlenecks into actionable design guidelines, each constraint can be framed in terms of the particle‐, field‐, material‐, and sensing‐level choices required to preserve function under task‐relevant conditions. Energy delivery therefore requires matching particle response and field timescales while minimizing parasitic dissipation; material compatibility requires preserving field‐responsive interfaces while maintaining stability and task‐specific chemistry; and sensing requires resolving the state variables needed for feedback with sufficient spatial and temporal fidelity. These design requirements can be evaluated through task‐relevant metrics such as actuation efficiency and the operating field range, retention of field responsiveness, mobility, and task‐specific selectivity in complex media, and localization or state‐estimation accuracy during closed‐loop operation. Below, we discuss these three constraints individually and outline potential research directions for addressing them.

**FIGURE 4:**
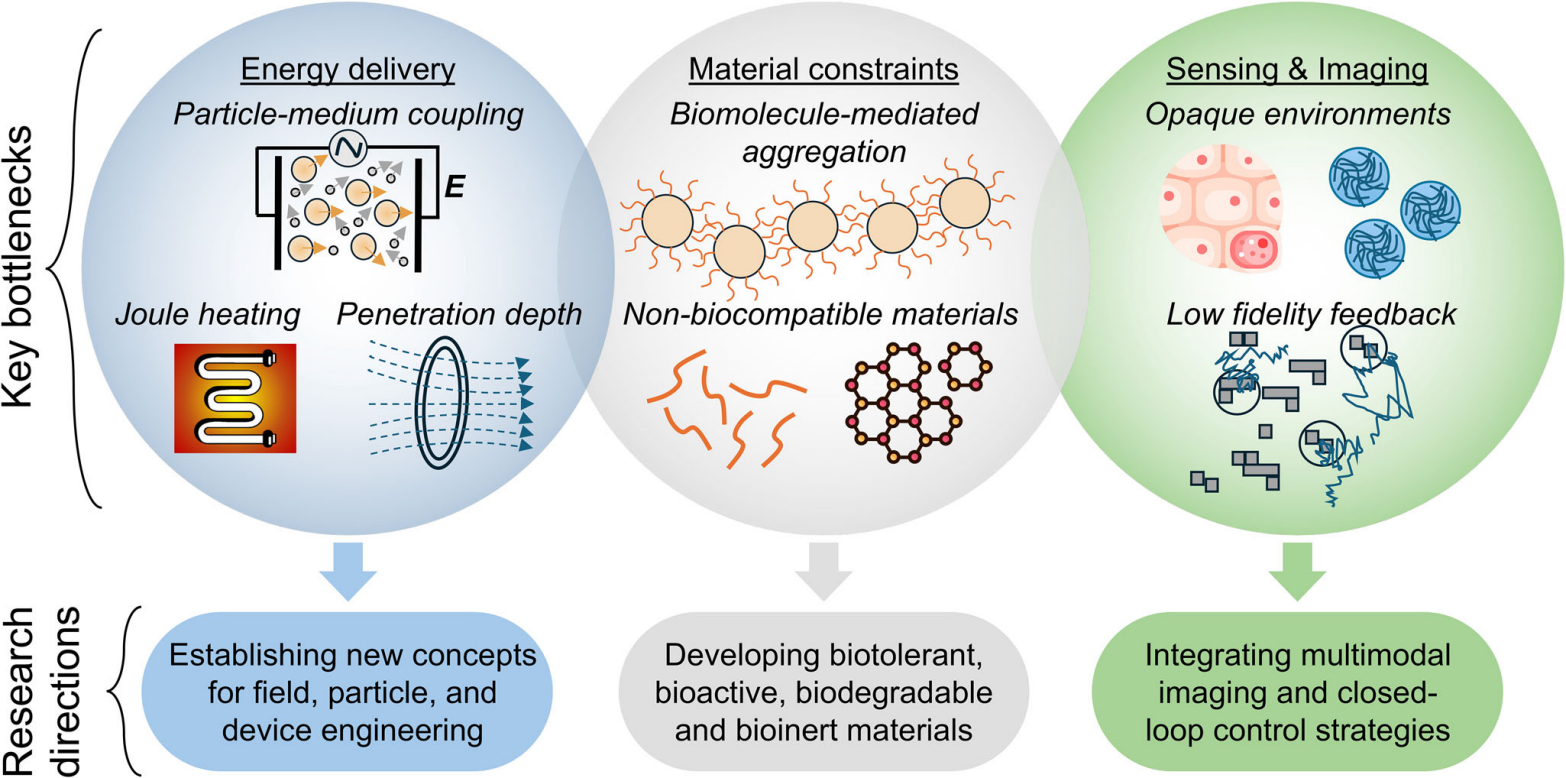
Key bottlenecks (top) and future research (bottom) for the translation of field‐driven colloids into functional micromachines. Energy delivery is limited by particle‐medium coupling, Joule heating, and field penetration depth, requiring field and device engineering for efficient actuation. Material constraints arise from biomolecule‐mediated aggregation and non‐biocompatible components, motivating biocompatible and antifouling material design that preserves function in complex media. Sensing and imaging constraints, including opaque environments and low‐fidelity feedback, require multimodal imaging and closed‐loop strategies that couple imaging, algorithms, and field modulation. Together, these bottlenecks define the design requirements for translating field‐driven colloids beyond proof‐of‐concept demonstrations toward task‐oriented micromachines.

Energy delivery remains a central bottleneck because external fields must couple strongly to actuate colloidal particles effectively while minimizing undesired perturbations to the surrounding medium and operating environment. In electric field‐driven colloids, the applied field acts not only on the particle but also on ions and interfaces in the suspending fluid, making propulsion sensitive to conductivity, ionic strength, frequency, and medium composition [15, 17, 19]. This constraint is especially important under AC fields, where the polarity‐switching timescale set by the driving frequency competes with the characteristic timescales of double‐layer charging, ion migration, interfacial polarization, electrohydrodynamic flow development, Brownian relaxation, and particle reorientation. When these timescales are mismatched, the applied field can be screened, dissipated into the medium, converted into parasitic flows, or coupled inefficiently to particle motion rather than producing controlled actuation [9, 49, 58]. Joule heating further constrains operation in conductive biological fluids, saline water, and environmental samples, where field application can change local temperature and alter both particle mobility and medium properties.

Contactless magnetic fields provide one route to overcome several limitations of electric actuation by enabling remote propulsion without directly driving ionic currents through the medium. Magnetic actuation, however, introduces its own coupling constraints because particle motion depends on the alignment, relaxation, and reorientation of magnetic moments relative to the applied field. For rotating or oscillating magnetic fields, the field modulation timescale must be tuned based on Brownian rotational diffusion, viscous relaxation, magnetic relaxation, and field‐induced torque response of the particle. When the driving frequency exceeds the ability of the particle or its magnetic moment to follow the field, phase lag and loss of synchronization lead to reduced propulsion efficiency [12, 15, 38, 47, 52, 53, 54, 58, 59, 60, 61]. Ferromagnetic particles with large magnetic moments can enhance magnetic responsiveness, whereas multicoil arrays and optimized field geometries can generate stronger and more directional fields over larger working volumes [62, 63]. Ultra‐low‐frequency magnetic fields can improve tissue penetration, and magnetophoretic guidance can use field gradients to bias particle transport toward deeper or less accessible regions [62, 64]. At the same time, strong field gradients and large magnetic moments can increase aggregation, wall attraction, off‐target accumulation, or nonspecific retention in heterogeneous media. These strategies improve field delivery but also introduce new requirements in particle design, instrumentation, scalability, and safety. Accordingly, future studies should evaluate not only propulsion speed, but also the field range over which controlled motion is maintained, the efficiency of field‐to‐motion coupling, and the extent of undesired heating, screening, or off‐target actuation under application‐relevant conditions.

Material compatibility determines whether field‐driven colloids can retain propulsion and function in complex biological, environmental, and materials‐processing media. A critical challenge is that fouling and adsorption do not merely block particle surfaces; they can alter how the applied field couples to the particle [15, 65]. In electric‐field‐driven colloids, adsorbed proteins, natural organic matter, surfactants, or polyelectrolytes can shift the slip plane, modify charge regulation, and alter Maxwell–Wagner relaxation times. These changes can lead to unpredictable alterations in dielectrophoresis, electrohydrodynamics, and induced charge electrophoresis, causing active colloids to show unpredictable kinematics or become trapped in complex media [13, 16, 19, 53]. In magnetic colloids, adsorbed biomolecules or polymer layers can increase drag, alter rotational relaxation, promote clustering, and shift the torque balance required for synchronization with rotating or oscillating fields. These field‐coupling changes manifest differently in biological and environmental media, where the dominant sources of interfacial modification arise from distinct molecular and ionic components [65, 66].

In biological fluids, proteins and other biomolecules can adsorb onto particle surfaces, promote aggregation, change cell interactions, or suppress field‐driven motion when surfaces are not appropriately functionalized [67, 68]. Non‐biocompatible or nondegradable components create additional barriers, particularly for drug delivery, where persistent or toxic materials may produce unintended biological responses rather than therapeutic function. Similar challenges arise in environmental media, where natural organic matter, pH variation, salinity, and competing ions can shift surface speciation, compress electrostatic double layers, alter ion mobility, bridge particles through multivalent interactions, and compete with target pollutants or ions for binding sites [65, 69]. Addressing these biological and environmental constraints requires materials that not only tolerate complex media but also preserve the field‐responsive properties required for actuation. Emerging materials strategies can be grouped into biotolerant, bioactive, biodegradable, and bioinert or antifouling approaches [70, 71, 72, 73, 74, 75]. Biotolerant and bioinert coatings, including hydrogels, polyethylene glycol (PEG), zwitterionic polymers, silica shells, and non‐fouling brushes, can suppress nonspecific adsorption and preserve particle mobility [76, 77, 78]. Bioactive designs, such as enzyme‐functionalized surfaces, cell‐membrane coatings, adhesive polymers, or chemically responsive ligands, can instead exploit local chemical or biological cues to enhance binding, localization, degradation, or release [79, 80]. Biodegradable matrices, including poly(lactic‐co‐glycolic acid (PLGA), polycaprolactone, chitosan, gelatin, alginate, and related polymeric materials, provide a route for particles to break down after completing a task [67, 79, 81]. Material design must therefore balance competing requirements: preserving the interfacial asymmetry needed for propulsion, maintaining colloidal stability in multicomponent media, minimizing nonspecific adsorption, and enabling task‐specific interactions such as cargo binding, pollutant capture, degradation, or release. A useful design criterion is therefore whether the particle retains its intended field response, colloidal stability, and task‐specific binding or release function after exposure to the relevant biological or environmental medium.

Sensing and imaging constraints determine whether field‐driven colloids can be observed, localized, and regulated with sufficient feedback during operation [13, 82, 83, 84]. Optical microscopy enables high‐resolution tracking in transparent laboratory media, but biological tissues, porous media, and opaque environmental samples restrict direct visualization. Recent optical‐clearing strategies may help address this limitation; for example, topical tartrazine was shown to reversibly increase tissue transparency in mice by reducing refractive‐index mismatch and light scattering [85]. The challenge is not only seeing the particle but resolving the particle state that is relevant for control, including position, orientation, speed, local environment, cargo state, and collective configuration. Multimodal imaging strategies, including optical coherence tomography, ultrasound imaging, magnetic resonance imaging, photoacoustic imaging, and fluorescence tracking, can extend observation into more complex environments, although each method involves trade‐offs in depth, resolution, contrast, speed, and material compatibility [65, 84, 86, 87, 88, 89, 90, 91]. Feedback fidelity presents an additional limitation because delayed, noisy, or incomplete localization can cause the control input to act on a measured particle state that differs from the actual trajectory or configuration. This mismatch becomes especially important for active colloids because Brownian fluctuations, boundary interactions, and collective rearrangements can change particle motion on timescales comparable to image acquisition and field modulation. Early demonstrations of closed‐loop control, cargo transport, and field‐guided navigation show how imaging, feedback algorithms, and field modulation can be integrated, but broader incorporation of model‐assisted control and AI/ML‐guided decision‐making remains needed [26, 28, 92, 93]. Progress should therefore be evaluated by whether the imaging modality can resolve the control‐relevant state with sufficient localization accuracy, temporal resolution, and robustness to support closed‐loop actuation in the intended environment. Overcoming energy, material, and feedback constraints will therefore be essential for transforming field‐driven colloids into reliable micromachines, motivating a shift from broad applications toward clearly defined functional tasks discussed in the following section.

## 5 Functional Tasks in Field‐Driven Active Colloids

Field‐driven colloids are beginning to show that externally controlled motion can be converted into task‐oriented function. Initial studies have established field‐driven propulsion and guidance as experimental capabilities, whereas current efforts increasingly ask what such motion enables particles to accomplish [15, 95, 96]. Electric and magnetic fields are especially important in this transition because they provide wireless, externally programmable, and fuel‐free control over particle motion. A task‐oriented view presented here therefore shifts the emphasis from broad application areas to the specific functions that field‐driven colloids must execute, including navigation, localization, transport, capture, treatment, recovery, and reconfiguration. Here, we categorize these emerging demonstrations into three broad classes of functional behavior: (1) Targeted navigation and delivery; (2) Selective uptake and recovery; and (3) Collective organization and reconfiguration (Figure 5). These classes are not restricted to a particular application domain. For example, navigation and transport are relevant to both biomedical delivery and environmental sampling, while capture and release can be critical in therapeutic delivery, pollutant removal, or materials processing. This task‐based organization therefore emphasizes the recurring functional operations that field‐driven colloids must execute and provides a more general framework for comparing micromachines across otherwise distinct applications.

**FIGURE 5:**
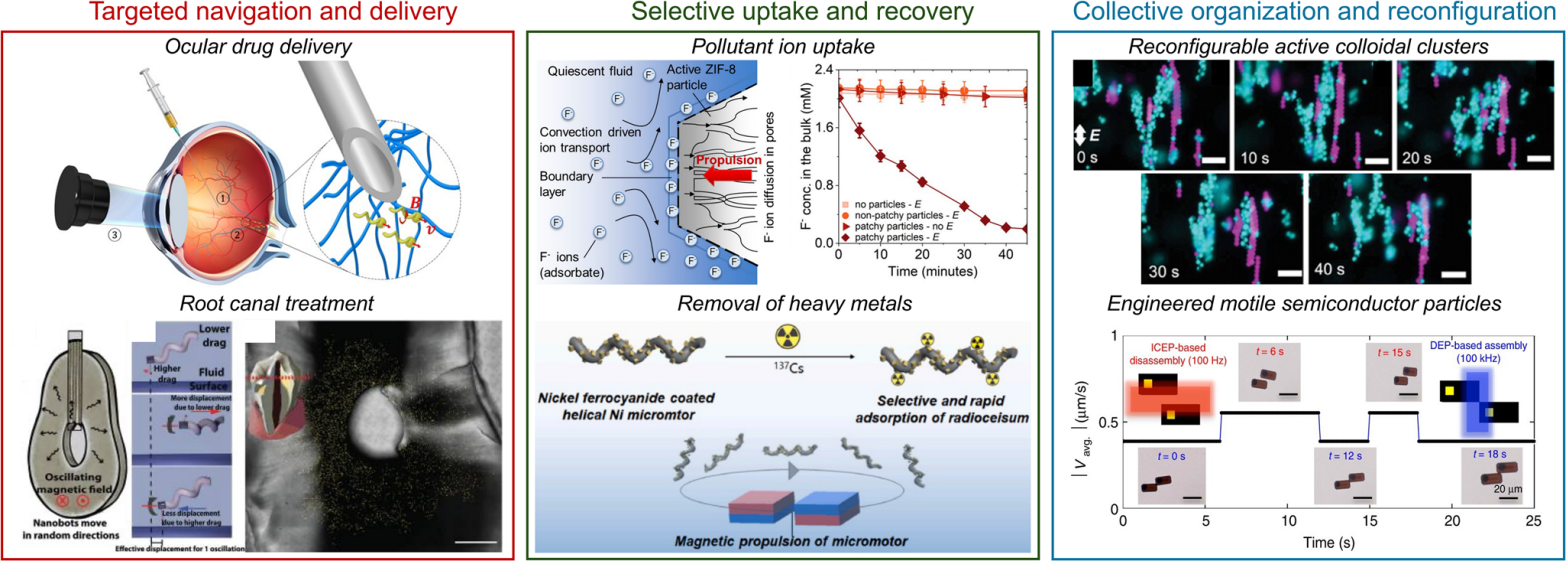
From applications to tasks in field‐driven active colloids. Field‐driven colloids can convert externally controlled motion into task‐oriented functions across (1) targeted navigation and delivery, (2) selective uptake and recovery, and (3) collective organization and reconfiguration. Representative examples include magnetic navigation for ocular drug delivery and root canal treatment (adapted from references [88] – Copyright 2018 AAAS and [89] – Copyright 2022 Wiley‐VCH), propulsion‐enhanced pollutant ion uptake and heavy‐metal removal (adapted from references [69] – Copyright 2024 American Chemical Society and [94] – Copyright 2024 Elsevier), and field‐programmed reconfiguration and assembly‐disassembly of active colloidal clusters (adapted from references [40] – Copyright 2025 Wiley‐VCH and [57] – CC BY 4.0) These demonstrations show how motion can be coupled to localization, transport, capture, treatment, and reconfiguration.

A task‐oriented description requires performance metrics that extend beyond propulsion velocity or trajectory shape, as summarized in Table 1. For example, in navigation, relevant quantities include target‐reaching probability, localization error, transit time, and recovery after perturbation. For transport and delivery, performance should include delivery yield, any off‐target deposition, cargo retention, and release efficiency. For pollutant capture or remediation, adsorption rate should be considered together with selectivity, treated volume, energetic cost, and recovery of contaminant‐loaded particles. For reconfigurable materials, switching time, structural fidelity, reversibility, and resistance to repeated cycling provide more meaningful measures than particle velocity alone. More generally, task performance can be viewed as a trade‐off among accuracy, completion time, field or energy input, and failure probability. Establishing such task‐specific benchmarks would allow field‐driven colloidal systems with different propulsion mechanisms, materials, and control architectures to be compared on the basis of function rather than motion alone.

**TABLE 1:** Task‐oriented performance metrics for active colloidal micromachines, highlighting representative controlled quantities, primary measures of task success, and metrics for evaluating robustness.

Task	Controlled quantity	Primary metric	Robustness metric
Particle navigation	Position/path	Success probability, endpoint error	Recovery after perturbation
Cargo transport	Cargo position/state	Delivery yield	Off‐target deposition
Target capture	Bound target fraction	Capture efficiency/selectivity	False capture
Biomedical treatment	Local dose/effect	Treatment success/healing	Off‐target side effects
Environmental Remediation	Pollutant concentration	Removal rate/selectivity	Loaded‐particle recovery
Collective state reconfiguration	Structural order parameter	Switching time/error	Reversibility/fatigue

Targeted navigation and delivery represent a class of task‐oriented function in which field‐driven colloids must reach defined locations, traverse complex environments, and deliver or perform a localized action [67, 97, 98]. Active colloids for biomedical functions provide the clearest demonstrations of these capabilities, where the small size and field responsiveness of the particles enable minimally invasive transport and intervention within confined anatomical spaces. Magnetic fields are especially suitable for such applications because they can penetrate biological tissues and generate forces or torques without requiring chemical fuels. This principle has been demonstrated in ocular delivery using magnetically driven helical micro‐propellers designed to overcome slow passive diffusion and hindered transport through the dense vitreous humor [88]. In this demonstration, long‐range propulsion under a rotating magnetic field was combined with a slippery perfluorocarbon coating that reduced adhesion to the vitreous network, showing how actuation and surface design can be integrated to address energy‐delivery and material‐compatibility constraints. Optical coherence tomography further enabled visualization of in vivo localization near the retina. A complementary demonstration in root canal treatment showed that magnetically maneuvered particles can access the narrow and tortuous dentinal tubule network, where residual bacteria often persist beyond the reach of passive antimicrobial delivery [89]. By coupling rotational and translational motion under magnetic fields, the active particles penetrated deep into dentine, distributed through the tubule network, and were recovered from selected regions. Additionally, the iron‐containing magnetic layer provided localized hyperthermia‐based antimicrobial activity. These studies show that biomedical function begins to emerge when field‐driven actuation, surface engineering, imaging, and multifunctional materials are integrated to overcome transport, adhesion, localization, and therapeutic barriers. Beyond application‐specific demonstrations, adaptive navigation in complex and crowded environments represents a more general functional challenge for field‐driven micromachines. A recent study further demonstrates how adaptive navigation can emerge without externally implemented sensing or feedback algorithms [99]. Flexible chains of concatenated active units driven by AC electric fields couple their conformation to their propulsion direction, generating an embodied mechanical feedback that enables autonomous reorientation at boundaries, navigation through obstacle arrays, collision avoidance, and continued motion through crowded environments. Such internally coupled sense‐response behavior provides a complementary route toward adaptive field‐driven micromachines in which environmental interactions directly modify locomotion. More broadly, future studies on electric‐ and magnetic‐field‐driven colloids should build on pre‐programmed navigation by integrating feedback control as a core design criterion. This could enable these micromachines to adapt to tissue heterogeneity, regulate therapeutic action, and report task completion in real time.

Selective uptake and recovery represent a class of task‐oriented function in which field‐driven motion is coupled to selective interactions with molecular, ionic, or particulate targets. Environmental remediation provides a useful demonstration of this task class because efficient treatment requires not only target binding, but also accelerated mass transport, chemical selectivity, and recovery of the contaminant‐loaded particles [65]. Field‐driven colloids are attractive for water remediation because adsorption‐based pollutant removal is often limited by diffusive transport to active binding sites, particularly at micron and submicron length scales. The key task in remediation is therefore not simply pollutant binding but accelerating transport across the particle‐scale boundary layer while preserving chemical selectivity and enabling recovery after capture. Electric and magnetic fields can supply fuel‐free propulsion and enhance interfacial mass transfer without introducing additional chemical fuels. This principle has been demonstrated using gold‐patched zeolitic imidazolate framework‐8 (ZIF‐8) particles, a porous metal‐organic framework (MOF) driven by AC electric fields through ICEP [69]. In this demonstration, the ZIF‐8 particles provided anion‐adsorption functionality, while propulsion accelerated the uptake kinetics of perchlorate, sulfide, and fluoride, showing how porous adsorbents can be integrated with externally powered motion for pollutant removal. Although the exact origin of the enhanced pollutant uptake at low Péclet number remained unclear, the result suggests that field‐driven motion couples near‐particle transport with diffusion and binding inside the porous MOF framework. A complementary magnetic‐field‐driven approach used helical nickel particles coated with cesium‐selective nickel ferrocyanide for radio‐cesium removal from water, where magnetic gradients and rotating magnetic fields generated pulling and tumbling modes [94]. The tumbling motion enhanced cesium adsorption kinetics by approximately 21‐fold relative to static conditions, while the nickel ferrocyanide coating provided high selectivity for radio‐cesium, illustrating how magnetic actuation can be coupled with pollutant‐specific binding chemistry. This example shows that efficient and selective remediation requires the integration of particle motion, rotation, near‐surface mixing, selective coordination chemistry, and magnetic recoverability. These studies show that field‐driven colloid‐based remediation is beginning to move beyond passive adsorption by integrating fuel‐free actuation, chemically selective binding, propulsion‐enhanced mass transfer, and magnetic recovery into a single platform. The next challenge is to make these particles adaptive rather than merely mobile. Future field‐driven colloids will need to sense pollutant gradients, regulate motion based on adsorption saturation, identify uptake/treatment endpoints, and enable scalable recovery of contaminant‐loaded particles. Achieving these capabilities would shift the field‐driven remediation from externally actuated adsorbents toward environmental micromachines that can locate, capture, concentrate, and remove contaminants in chemically heterogeneous water.

Collective organization and reconfiguration define a class of task‐oriented functions in which field‐driven colloids are used to program the structure and collective state of matter itself [31, 56, 95, 100]. Here, the objective is not necessarily to reach or transport an external target, but to use field‐controlled interactions and propulsion to form, erase, or continuously reorganize nonequilibrium structures. Such reconfigurable active materials raise a different scientific question from transport or delivery: *how can field inputs select, maintain, and erase non‐equilibrium structural states stabilized by attraction, propulsion, and dissipation?* This concept has been demonstrated in active‐passive colloidal mixtures, where AC electric fields generate both dipolar attractions and ICEP‐mediated hydrodynamic flows, producing motile clusters that retain cohesion while undergoing continuous splitting, merging, and reorganization [40]. The degree of reconfiguration can be tuned by the active‐particle fraction, which controls the magnitude and timescale of cluster‐number fluctuations, showing that the material's dynamic state can be programmed through composition. This example shows how reconfiguration emerges from a competition between attractive interactions that stabilize clusters and active flows that continually “remodel” them. A related demonstration used microfabricated semiconductor particles whose size, shape, polarizability, and patterned coatings encoded different responses to AC electric fields [40, 57]. By switching the field frequency, these particles transitioned between dielectrophoresis‐driven assembly at higher frequencies and induced charge electrophoresis‐driven disassembly at lower frequencies, demonstrating reversible, on‐demand restructuring of microscale assemblies [57]. The frequency‐dependent transition from equilibrium to non‐equilibrium states of the particles highlights that external fields can select different interaction pathways in the same material by changing how polarization, hydrodynamic slip, and particle geometry couple to the surrounding medium. More recently, this concept has been extended from field‐switched restructuring of few‐particle assemblies to closed‐loop control of large anisotropic colloidal ensembles [101]. By using feedback to switch between attractive and repulsive dielectrophoretic interactions, assemblies containing hundreds of rectangular particles were navigated through metastable and glassy configurations toward nearly defect‐free crystalline states. This demonstration shows that external fields can be coupled with feedback not only to trigger assembly and disassembly, but also to actively navigate complex collective‐state landscapes and correct structural defects during reconfiguration. Field‐driven colloids can therefore function as energy‐dissipating building blocks for adaptive materials whose structure is not fixed after assembly but continuously remodeled by externally controlled activity.

The task‐oriented view presented above shows how synthetic field‐driven colloids are moving beyond demonstrations of microscale motion toward functional behaviors that couple field‐directed propulsion to therapeutics, pollutant capture, and material reconfiguration. However, most field‐driven colloids remain semi‐autonomous because actuation is externally prescribed and task progress is evaluated outside the particle [19, 65]. Future reconfigurable micromachines will require feedback mechanisms that link structural state, field input, and desired material response, enabling assemblies that not only form and break apart on demand but also adapt their organization, mechanical/transport properties, or optical response in real time.

## 6 Toward Autonomy: Limits of Scaling and the Role of Feedback

Field‐driven colloids have begun to progress from demonstrating motion to executing functions, but most demonstrations remain semi‐autonomous rather than truly adaptive [13, 15, 103]. In many cases, the applied field is prescribed externally, while particle position, environmental features, and task completion are measured separately from the actuation process. Such open‐loop operation enables controlled motion but does not allow the micromachine to correct errors, respond to disturbances, or update behavior as the task evolves. The autonomy gap therefore lies not in making each particle independently intelligent, but in closing the loop among sensing, computation, and field‐based actuation.

It is useful to distinguish between closing the feedback loop on motion and closing it on the desired function. Most current demonstrations of autonomous or feedback‐guided microrobotic behavior regulate variables that are readily obtained from imaging, including particle position, orientation, trajectory error, swarm morphology, or obstacle location. Such approaches can achieve highly sophisticated navigation while the functional state of the micromachine remains effectively open loop. For example, a particle may autonomously reach a target without determining if its cargo was successfully released, if contaminant adsorption sites were fully saturated, or is a desired material reconfiguration has been completed. The next stage of field‐driven active colloids will require feedback architectures in which functional variables to become part of the measured state used to determine subsequent field inputs. These functional variables may include the state of bound cargo, local contamination or treatment progress, or collective structural state. We refer to this distiction as “trajectory‐closed” vs “task‐closed” control.

Autonomy at colloidal length scales should be viewed differently from autonomy in macroscopic robots because sensing, computation, power, and actuation cannot usually be integrated within each particle [11, 104]. Instead, autonomy must be externalized across the particle, imaging modality, control algorithm, and applied field. Feedback control provides the architecture for this distributed autonomy in which image acquisition and particle tracking define the instantaneous state of the particle, a path‐planning or control module compares this state with a desired trajectory, and magnetic or electric fields are updated to reduce the error (Figure 6A,B). Recent work with magnetic microellipsoids demonstrated this principle by integrating real‐time particle and obstacle sensing, path planning, and magnetic field actuation to achieve controlled navigation through maze‐like environments without human intervention [26]. The same platform also showed cargo capture, transport, and release through field‐directed interfacial motion, illustrating how feedback can connect particle navigation to task execution.

**FIGURE 6:**
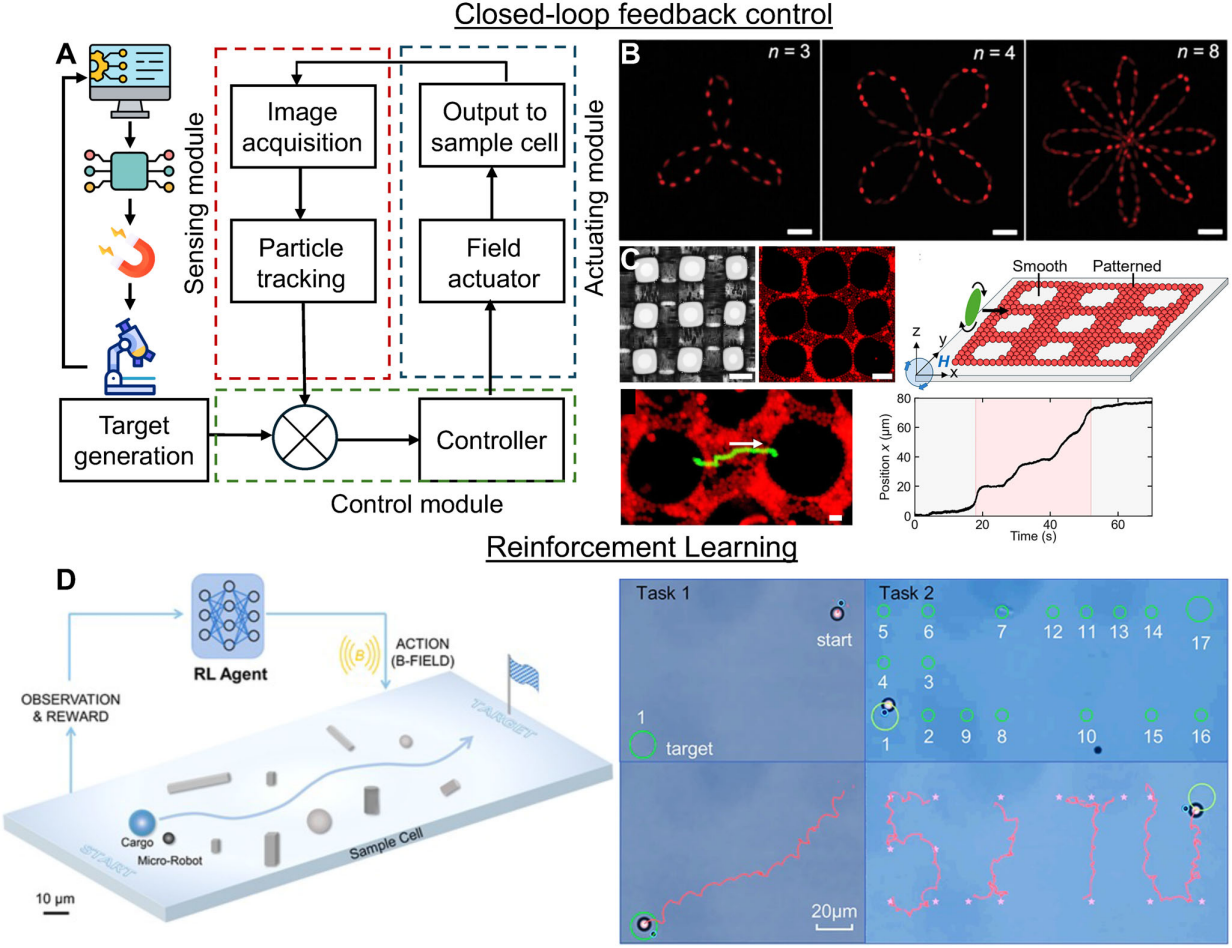
Feedback control as a route toward adaptive field‐driven micromachines. (A) Closed‐loop control architecture in which image acquisition and particle tracking define the particle state, a control module compares this state with a desired trajectory, and field actuation updates particle motion. (B) Feedback‐guided magnetic microllipsoids can be steered along programmed trajectories, demonstrating real‐time control over particle navigation. (C) Vision‐based feedback enables magnetic micro ellipsoids to detect and respond to heterogeneous surface topography, allowing locomotion across smooth and patterned regions. Panels B is adapted from reference [26] (Copyright 2024 Wiley‐VCH) and Panel C is adapted from reference [28] (Copyright 2026 Elsevier). (D) Reinforcement‐learning‐based feedback control of a magnetic colloidal microrotor for contactless cargo transport, integrating real‐time visual tracking with learned field actions to achieve target‐directed delivery and multi‐step navigation. Panel D is adapted from reference [102] (Copyright 2026 CC BY 4.0).

Scaling feedback from single‐particle navigation to collective micromachine operation introduces a fundamental control problem because collective behavior cannot be treated as a simple extension of single‐particle motion [14, 15, 18, 53, 105, 106]. A global electric or magnetic field can efficiently drive many particles at once, but this same global coupling makes it difficult to address each particle independently. As particle number density increases, particle‐level properties such as size, geometry, polarizability, magnetic moment, surface charge, propulsion speed, rotational mobility, and interaction anisotropy determine how particles organize under the applied field [13, 15]. Many‐body hydrodynamic interactions, dipolar attractions, steric collisions, wall effects, field‐induced aggregation, and medium‐mediated flows can amplify small particle‐level differences, producing clusters, chains, swarms, vortices, jams, or phase‐separated regions [13, 16, 53]. The central scientific challenge is to determine how particle design and field parameters map onto collective observables such as cluster size, particle density, orientation distribution, transport flux, spatial coverage, and reconfiguration rate. Established order parameters and microstructural descriptors from soft‐matter physics provide a natural reduced representation of these many‐particle states by quantifying positional, orientational, and dynamical order in physically interpretable terms. Such descriptors can therefore provide the state variables needed to connect particle‐scale interactions and field inputs to emergent collective behavior. For systems exhibiting highly heterogeneous or dynamically reconfiguring states that are difficult to capture using a small number of predefined order parameters, data‐driven dimensionality‐reduction methods can complement these physics‐based descriptors by identifying additional collective coordinates directly from experimental data [107, 108]. Future control strategies will therefore require a combination of physically informed collective variables, data‐driven models, and feedback laws that regulate collective organization toward desired functional outcomes.

Current feedback‐guided field‐driven colloids remain limited by sensing fidelity, environmental complexity, and globally coupled actuation [20, 109]. Imaging depth, contrast, and frame rate determine how accurately particle position or collective state can be measured. Noisy localization and time delays can cause field updates to act on outdated particle states. Heterogeneous surfaces and confined geometries introduce additional disturbances [23, 90, 110, 111, 112]. Recent work showed that vision‐based feedback can recognize surface topography in real time and adapt magnetic field parameters to guide microellipsoids across smooth and patterned regions [28] (Figure 6C). Additionally, reinforcement learning has begun to extend closed‐loop control from trajectory correction toward adaptive decision‐making and task planning [113]. A study combined real‐time visual tracking with a reinforcement‐learning agent to control a magnetic colloidal microrotor for contactless transport of nonmagnetic cargo (Figure 6D) [102]. The agent selected magnetic field‐driven actions based on the instantaneous positions of the rotor, cargo, and target, enabling target‐directed delivery, while progressive training and path‐planning strategies further enabled obstacle avoidance and maze navigation. The agent was trained in a simplified simulated environment and subsequently implemented experimentally in the presence of Brownian motion, hydrodynamic perturbations, and finite feedback latency. This study demonstrated the potential for learned control policies to transfer from simulation to experimental colloidal platforms. The broader challenge is to make feedback predictive rather than only corrective. AI, machine learning, and data‐driven models can help learn particle response maps, infer hidden states, and select field inputs that improve task progress in real time [54, 114]. Near‐term progress will require model‐assisted feedback, adaptive field modulation, and data‐driven control strategies that connect sensing, actuation, and functional outcomes. Long‐term progress will require adaptive feedback architectures that learn from prior trajectories, infer hidden states such as adhesion, local drag, or chemical gradients, and autonomously update control policies as micromachines operate in changing environments [103, 115, 116, 117, 118]. The sensing challenge is therefore not only to localize particles more accurately, but to convert otherwise hidden chemical, interfacial, mechanical, or cargo states into experimentally accessible feedback signals.

## 7 Outlook: A Control‐Centric Roadmap

Field‐driven active colloids occupy an intermediate regime where fluctuations, interactions, and external control can be coupled to generate programmable motion across scales [13, 15, 17, 19]. By framing these particles as distributed micromachines, this Perspective highlights how power delivery, sensing, control, and function can be supplied through an external particle‐field‐imaging‐controller architecture rather than integrated within each particle. Major advances in field‐driven motion, navigation, and assembly have established active colloids as promising micromachine building blocks. Realizing multimodal task‐oriented function, however, will require parallel progress in fundamental science and control architecture. A deeper understanding of the physics of particle‐field‐medium interactions, interfacial coupling, and collective dynamics must be integrated with feedback‐guided control frameworks that convert motion into adaptive, measurable, and application‐relevant behavior.

Although prior studies have advanced field‐driven active colloids through propulsion design, trajectory programming, collective dynamics, and feedback‐guided navigation, most discussions have treated these advances as separate developments in active matter, microrobotics, or application‐specific demonstrations [15, 17]. This Perspective takes a different view by unifying these efforts under a distributed micromachine framework, where particles, fields, imaging, feedback control, and task‐specific materials together form the functional architecture needed to bridge physics, control, and function across scales. The next stage of field‐driven colloids should be guided by a control‐centric roadmap that integrates propulsion, sensing, feedback, computation, collective coordination, and function. Physics and propulsion remain the foundation, because particle shape, composition, anisotropy, field strength, frequency, and boundary interactions determine how motion and interactions are generated. Sensing and imaging must then provide the information needed to observe particle position, orientation, collective state, and environmental features during operation. Feedback control closes this loop by comparing the measured state with a desired outcome and updating the applied field to minimize error. From this perspective, autonomy should not be treated as an intrinsic property of isolated particles, but as an emergent capability of a coupled particle‐field‐imaging‐controller architecture (Figure 7).

**FIGURE 7:**
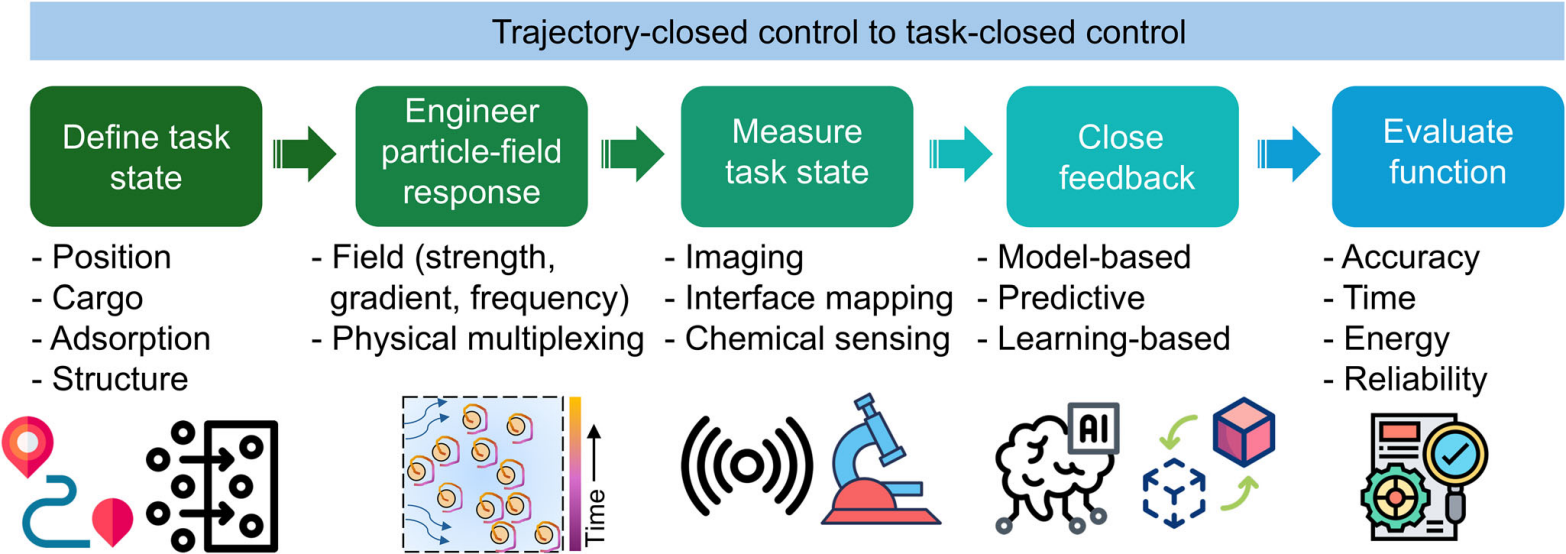
Control‐centric design framework for distributed micromachines. Functional micromachine design begins by defining a reduced set of task‐relevant state variables, followed by engineering particle‐field responses that enable these variables to be regulated through externally applied field(s). Imaging and other sensing modalities then resolve the task‐relevant states with high spatial and temporal fidelity for feedback. Model‐based, predictive, or learning‐based controllers can update field inputs to minimize deviations from the desired task state. Performance should ultimately be evaluated through functional metrics such as accuracy, completion time, control effort, reliability, and recovery from perturbations rather than particle motion alone. This framework shows a progression from trajectory‐closed and environment‐responsive control toward task‐closed control, in which feedback directly regulates functional outcomes such as cargo delivery, pollutant capture, treatment progress, or collective reconfiguration.

Functional tasks provide the most useful benchmark for evaluating progress in field‐driven active colloids. Rather than emphasizing motion alone, future studies should quantify whether field‐driven colloids can localize, transport, penetrate, capture, treat, release, assemble, or reconfigure matter with measurable efficiency, accuracy, and reliability. Collective coordination represents the next level of this challenge, because many practical functions will require populations of particles rather than a single active colloid. Coordinated ensembles could improve pollutant capture, cargo distribution, local treatment, or macroscale mechanical reconfiguration, but they also require strategies for controlling collective motion, density, organization, and recovery. This task‐based framing shifts the emphasis from demonstrating individual propulsion toward quantifying and programming functional outcomes across many interacting colloids.

The above considerations suggest a potential sequence for designing future field‐driven distributed micromachines:

1. The task must be expressed through a small number of physically meaningful state variables rather than through a broad application label.
2. The particle and field architecture should be designed so that these variables respond reproducibly and independently to available field parameters.
3. Sensing must resolve the same task‐relevant variables on a timescale faster than their uncontrolled evolution.
4. Feedback should be evaluated by whether it improves task completion rather than only particle‐level response latency or trajectory accuracy.

This sequence of defining the task state, establishing actuation, observability, and then closing the loop provides a practical design roadmap connecting colloidal physics with control and function.

AI and data‐driven control should be viewed as complementary rather than replacements for physical understanding of field‐driven colloids. Recent machine learning‐based microrobotic studies have already demonstrated model‐free trajectory control, path planning, obstacle avoidance, swarm‐state regulation, simulation‐to‐experiment transfer, and decision‐making under incomplete observations [119, 120, 121, 122]. These advances show that machine learning can be particularly valuable when particle‐field‐environment coupling is difficult to model explicitly or changes during operation. However, important limitations remain. Model‐free reinforcement learning can require large training datasets and extensive exploration, while policies trained under one particle, medium, imaging configuration, or device geometry may not be applicable to another [123, 124]. Learned controllers can also be nontrivial to interpret, which may fail under conditions outside their training distribution [125]. Such controllers are not usually informed by functional or task‐focused variables such as cargo release, chemical binding, adsorption saturation, or treatment completion. Near‐term progress is likely to come from physics‐informed and model‐assisted strategies. Such approaches may combine experimentally constrained particle dynamics with state estimation, predictive control, and machine learning‐based adaptation. The important transition is not simply from conventional control to AI, but from controllers that optimize motion to infer hidden physical states and optimize task completion under uncertainty. More broadly, the decisive advance in the area will not be the miniaturization of every component of a macroscopic robot, but the ability to identify, observe, and control the small number of collective and functional variables required for a microscopic task.

## Author Contributions

**Ruchi Patel**: conceptualization, writing – review and editing; **Bhuvnesh Bharti**: conceptualization, funding acquisition, supervision, writing – review and editing.

## Conflicts of Interest

The authors declare no conflicts of interest.
